# Decision-making about breastfeeding: influences of breastfeeding counseling

**DOI:** 10.1590/0034-7167-2024-0599

**Published:** 2026-01-09

**Authors:** Jéssica Aparecida da Silva, Monika Wernet, Allison Scholler de Castro Villas Boas, Elisa da Conceição Rodrigues, Karine Emanuelle Peixoto Oliveira da Silva, Mariana Torreglosa Ruiz

**Affiliations:** I Universidade Federal do Triângulo Mineiro. Uberaba, Minas Gerais, Brazil.; II Universidade Federal de São Carlos. São Carlos, São Paulo, Brazil.; III Universidade Federal do Rio de Janeiro. Rio de Janeiro, Rio de Janeiro, Brazil.; IV Universidade Estadual de Feira de Santana. Feira de Santana, Bahia, Brazil.

**Keywords:** Counseling, Decision Making, Breast Feeding, Weaning, Qualitative Research, Consejo, Toma de Decisiones, Lactancia Materna, Destete, Investigación Cualitativa

## Abstract

**Objectives:**

to understand how women who received breastfeeding counseling make breastfeeding-related decisions during their children's first year of life.

**Methods:**

a qualitative study conducted with women who received breastfeeding counseling in rooming-in units of three university hospitals, two located in the Southeast region and one in the Northeast region. Semi-structured interviews were conducted between May and September 2024, assessed using the Symbolic Interactionism and Thematic Analysis frameworks.

**Results:**

eleven women participated, highlighting the interactional context of breastfeeding counseling as a promoter of safety and trust, with technical and emotional contributions to overcoming breastfeeding challenges.

**Final Considerations:**

breastfeeding counseling proved to be a care technology with positive influences on maintaining exclusive breastfeeding in the first year of children's lives.

## RESUMEN

**Objetivos:**

comprender cómo las mujeres que recibieron consejería en lactancia materna toman decisiones relacionadas con la lactancia durante el primer año de vida de sus hijos.

**Métodos:**

estudio cualitativo realizado con mujeres que recibieron consejería en lactancia materna en unidades de alojamiento conjunto de tres hospitales universitarios, dos ubicados en la región sureste y uno en la región noreste. Se realizaron entrevistas semiestructuradas entre mayo y septiembre de 2024, evaluadas mediante los marcos de interaccionismo simbólico y análisis temático.

**Resultados:**

once mujeres participaron, destacando el contexto interaccional de la consejería en lactancia materna como promotor de seguridad y confianza, con contribuciones técnicas y emocionales para superar los desafíos de la lactancia materna.

**Consideraciones Finales:**

la consejería en lactancia materna demostró ser una tecnología de cuidado con influencias positivas para mantener la lactancia materna exclusiva durante el primer año de vida de los niños.

## INTRODUCTION

Although evidence supports the recommendation for breastfeeding and the benefits of human milk^(1)^, less than half of children are exclusively breastfed until the sixth month of life^(2)^, a rate similar to the prevalence of exclusive human breastfeeding until six months of age in Brazil^(3)^. These data demonstrate high and alarming weaning rates worldwide as well as among Brazilian children^(4,5)^.

The World Health Organization (WHO) indicates and recognizes breastfeeding counseling as a technology for preventing and promoting breastfeeding^(4)^. A systematic review study with meta-analysis supported counseling as an effective public health intervention for increasing breastfeeding duration rates, including exclusive breastfeeding^(6)^. Breastfeeding counseling transcends clinical management and guidelines, being structured in the horizontal and dialogical relationship between the professional (counselor) and the person who breastfeeds or intends to breastfeed, based on Carl Rogers’ Client-Centered Therapy^(7)^. Its proposal is to support breastfeeding, with consideration and respect for the particularities and desires of women who breastfeed or intend to breastfeed^(4,5)^.

Professionals are not responsible for determining what and how a woman should do, but they should provide support so that she can make decisions that take into account the unique aspects of her experience^(5)^. To this end, professionals should undergo training in breastfeeding counseling, integrating theoretical and practical aspects. This training aims to develop complex communication skills in counselors based on the knowledge and skills for breastfeeding support, aligned with the project, reality, and possibilities of each woman and family^(4,5,8)^. Its pillar is the centrality of the relationship between the professional and the person being counseled, with investments in listening skills, valuing empathy and praise, and discouraging judgment, with a view to increasing the confidence of women and family in the act of breastfeeding^(4,5,8)^.

This is a randomized clinical trial carried out with 102 primiparous women, in which counseling sessions on human breastfeeding were applied during the dyad’s hospitalization in rooming-in (RI), compared to usual care, indicated that women who received the intervention were more likely to maintain exclusive breastfeeding until the sixth month of life^(9)^, demonstrating the long-term reach of the strategy.

Along these lines, a qualitative study with a phenomenological approach conducted in Norway found that breastfeeding women were reflecting on the possibility of weaning when they received support through counseling, which was essential for them to maintain weaning, reestablish themselves, and continue breastfeeding. They emphasized that the approach helped them be seen both as breastfeeding mothers and as women with their own needs^(10)^.

The evidence presented, both quantitative and qualitative, demonstrates the impact of breastfeeding counseling provided by a trained professional on the decision-making process of women intending to breastfeed.

Given the high rates of early weaning, the potential repercussions for child health, and the need to expand knowledge about breastfeeding counseling due to the scarcity of studies on the topic, even with evidence of its effectiveness, the phenomenon under exploration was understanding the impact of breastfeeding counseling on breastfeeding decision-making that justifies its implementation.

## OBJECTIVES

To understand how women who have received breastfeeding counseling make decisions related to breastfeeding during the first year of their children’s lives.

## METHODS

### Ethical aspects

The study received ethical approval from the Research Ethics Committee, under Opinion 6,274,311, dated August 31, 2023 (Certificate of Submission for Ethical Consideration 4 61321122.3.1001.8667), using guidelines and regulatory standards for research involving human subjects contained in National Resolution 510/2012/CNS/MoH. It also complied with the ethical principles contained in the Declaration of Helsinki, ensuring that all study participants signed the Informed Consent Form.

### Study design

This is qualitative research, the development and reporting of which considered the COnsolidated criteria for REporting Qualitative research^(11)^.

### Theoretical-methodological framework

This study adopted Symbolic Interactionism (SI) as its theoretical and methodological framework, according to which people behave based on the interpretations and meanings established in interactional processes, both with themselves and with others^(12)^. Sense, meaning, and behavior are always open, subject to transformation, and dependent on what emerges from social interaction^(12)^.

The act of breastfeeding is influenced by the senses and meanings a person has about themselves, their body, food, the maternal role, breastfeeding, breastfeeding, and their relevance to themselves, their family, and society. Therefore, breastfeeding is a complex social practice that involves self-interaction and, concomitantly, expanded social interactions^(13)^.

### Methodological procedures

The participants in this study were part of an intervention group of a pilot study of a randomized clinical trial (RCT), developed in three centers, aimed at investigating the effectiveness of breastfeeding counseling during the hospitalization of the dyad (mother and her newborn) in RI, its impact on exclusive breastfeeding duration and maintenance^(14)^. The intervention group received two to four breastfeeding counseling sessions during their hospitalization in RI. Each center had a counselor, a registered nurse, trained in breastfeeding counseling (76 hours of theoretical and practical training). The choice of a nurse was based on the fact that the literature indicates that they are the professional most cited by women in actions to protect and promote breastfeeding^(15)^. It should be noted that, to avoid potential response bias, separate teams were formed for the different study objectives. Researchers who implemented the pilot RCT intervention were designated, and the researchers who conducted the interviews that comprise this study did not participate in the data collection for the intervention study.

Inclusion criteria for participation in the pilot RCT study were being primiparous women with an only live birth, gestational age of 37 to 42 weeks (term), and weight greater than 2,500 grams, regardless of the mode of delivery. Exclusion criteria were: postpartum women and newborns with contraindications for breastfeeding; newborns with malformations that prevented or hindered breastfeeding and/or with altered breastfeeding mechanics (lingual frenulum); postpartum women whose newborns were immediately separated after umbilical cord clamping at birth due to maternal-neonatal complications; postpartum women transferred from other institutions or who had already been discharged (readmitted) at the time of allocation; postpartum women who used illicit drugs or alcohol; and postpartum women with intellectual and/or sensory disabilities, based on a diagnosis recorded in the medical records.

For this study, all participants in the pilot study intervention group who reported having internet access and the necessary conditions and resources to conduct video calls were considered. The invitation to participate occurred during follow-up calls from the RCT pilot study, with reinforcement via written messages via WhatsApp®. If participants expressed interest in participating, an online Informed Consent Form was sent, and the interview was scheduled on a day and time of participants’ preference.

The interviews took place online via video calls on the WhatsApp® platform, with audio recordings mediated by a voice recorder. The interviews were conducted by two researchers: the first and third authors of this manuscript. Prior to data collection, both received training in qualitative interviews and the study framework (a total of 60 hours) from the second author, a researcher with expertise in developing qualitative studies. Furthermore, data collection and analysis occurred concurrently, always under the support and supervision of the third and final author, a factor that favored rich data.

Whether or not a participant used a camera was respected, but both interviewers kept theirs on, with the intention that facial and gestural communication, as well as other paralanguage elements, would contribute to establishing interactional comfort during data collection and communicate interest in the narrative. At the beginning of the video call, participants were asked for permission to record the video, and the purpose of the recording was clearly explained.

To counter the limitations of video call data collection, we have listed the procedural precautions adopted. To minimize the diversity of technology access, women had the option of keeping the camera on or off during the interviews, and all interviews were audio-recorded, so the quality of the technology did not interfere with the results. Since the interviews were not conducted in a controlled environment, where it is not possible to control ambient noise and speech, as well as distractions, which could lead to participant interruptions, we adopted the criterion of scheduling interviews at a time convenient for participants. The researchers were trained in communication skills to return to the point of uncertainty or resume after interruptions. Concerning the limitation of non-verbal language capture, it is worth noting that audio recording allowed for the identification of pauses and emotions in participants’ speech when the camera was off. Therefore, care was taken to overcome or minimize the limitations of the adopted strategy.

### Study setting

The RCT pilot study was conducted at three centers, in their respective RI units. These are public university hospitals located in Minas Gerais, Rio de Janeiro, and Bahia.

At the institution in Minas Gerais, the unit where the women were allocated consists of 12 individual RI wards, where the dyad and their companion remain together. The institution does not have the Baby-Friendly Hospital designation, although it is one of its goals. It is a reference for high-risk and normal pregnancies in its coverage area, and assists 27 municipalities, including those without a maternity ward. The institution has a standard operating protocol for breastfeeding assistance; however, the procedures only cover clinical breastfeeding management actions.

The Rio de Janeiro hospital offers outpatient, inpatient, and multidisciplinary care for high-risk pregnant women and newborns. The RI unit has nine wards, each with five beds, totaling 45 beds. The institution was awarded the Baby-Friendly Hospital designation in December 2020.

The Bahia hospital has been accredited as a Baby-Friendly Hospital since 1995. A reference for low-risk pregnancies, the public educational institution has three RI units with a combined capacity of 95 beds.

### Sample

A total of 29 potential participants (intervention group of the RCT pilot study) were selected for this research. However, three women were excluded because they lacked the resources and conditions to receive video calls, 12 because they did not respond after three attempts to contact the researchers, and three because they refused to participate, citing a lack of time to participate.

In the ninth interview, no new elements emerged, indicating data density. However, since two women remained to cover the total number of participants who indicated interest and availability to participate, interviews with them were conducted, demonstrating data saturation^(16)^.

### Data collection and organization

Data collection took place between May and September 2024. The interviews were audio-recorded, lasting an average of 30 minutes, and used a semi-structured script. The script included the following questions: how have you been feeding your child from birth to the present day? Recall your interactions with the breastfeeding counselor. What was significant to you? How did these interactions influence your decision-making regarding breastfeeding? Other questions aligned with the above were presented whenever it was necessary to expand understanding of the narrative.

The interviews were transcribed using the Transkriptor® application and transferred to a Word® document by the first author, and later checked in full by the second author. To protect participants’ identities, they were identified by the numerical order in which the interviews were conducted (e.g., P1, P2...), and the names mentioned during the interviews were replaced by the initial letter. It should be noted that the transcripts, after checking by the researchers, were sent via WhatsApp® to participants, with a request for adjustments, if necessary, which did not occur, and are therefore considered validated.

### Data analysis

The audio transcription documents were subjected to the thematic analysis process, proposed by Braun and Clarke (2006), using the Atlas.ti® software for grouping information. The analytical process, developed by the first, second, and third and last authors, went through six phases: familiarization with the data; generation of initial codes; search for topics; review of topics; definition and naming of topics; and production of report^(17)^. The other authors participated in meetings to discuss the analysis, providing contributions.

It is important to note that, during the analysis process, transcripts were actively read repeatedly, searching for meanings, including other latent information from participants’ experiences, in search of the underlying meaning of words. Concurrently, initial codes were generated from the transcribed narratives, which identified common characteristics of the data, giving rise to topics (the main and broader unit of analysis)^(17)^.

## RESULTS

### Participant characterization

Eleven women participated in the study, four of whom were treated at a hospital in Minas Gerais, four at a hospital in Rio de Janeiro, and three at a hospital in Bahia. The women had an average age of 27, ranging from 21 to 35. Eight women reported living with a partner, and three declared themselves single. Eight women self-identified as mixed race, all literate. One had completed elementary school, two had incomplete high school, six had completed high school, one had incomplete higher education, and one had completed higher education. Six women were employed, and their family income ranged from one to three times the minimum wage.

Regarding the mode of birth, five were delivered vaginally, while six underwent cesarean section. Children’s ages at the time of data collection ranged from 14 to 18 months, with an average of 15 months. All were breastfed at some point in their lives, with the majority (n=7) remaining exclusively breastfed for six months, and of these, six were still breastfeeding at the time of the interview.

The analysis identified three topics: “Presence behavior and care developments”; “Counseling and decision-making”; and “Counseling symbolisms” (Chart 1).

**Chart 1:** Topics, subtopics and codes from the study “Decision-making about breastfeeding: influences of breastfeeding counseling”, Uberaba, Minas Gerais, Brazil, 2024

Codes	Subtopic	Topic
Respect and availability	Implied presence	Presence behavior and care developments
Loving listening		
Mutuality		
Consideration when becoming a mother	Reception	
Consideration of breastfeeding possibilities		
Breastfeeding complications	Breastfeeding stakeholders	Counseling and decision-making
Nipples, bottles, and maintaining breastfeeding		
Social constraints and breastfeeding		
Incentives	Counselor support	
Memories of counseling		
Being a nursing mother and first-time mother	Acknowledgment	Counseling symbolisms
First-time mother and motherhood		
Technical tips	Partnership	
Emotional support		

Breastfeeding counseling emerges in the statements as a technical and emotional support for mothers, helping them make decisions and face the challenges of breastfeeding with greater security and confidence. However, in some situations, it was not sufficient to provide a counterpoint to participants, especially regarding family beliefs and values.

### Presence behavior and care developments

The women highlighted a nurse counselor’s presence as a distinguishing feature of their experience, conveying recognition, interest, and respect for them and the process of becoming mothers. This presence was permeated by affection, fostering an interactional process of mutuality and acceptance, with relational comfort. Women felt noticed, heard, valued, and supported in breastfeeding as well as in motherhood and emotional issues.

[...] *I felt very welcomed by her* [...] *it seemed like we had known each other for millions of years* [...] *the affection she had, the attention she gave me* [...] *because I had already gone through a very difficult time, which was my birth* [...] *so, she welcomed me with words of affection* [...] *I cried a lot* [...] *because I was depressed* [...] *so she sat next to me* [...] *she talked... she held [child’s name], so she helped me a lot.* (P2 – 21 years old, married, brown, completed high school)

*When she sat down and talked to me, and explained, it was a conversation. She didn’t come up to me and say, “Look, you have to do this, because this, that, something else”. I think it was the way she conducted the conversation, because we, especially when you’re there, and when you’re a first-time mother, I needed that real, verbal welcome, I needed them to look me in the eye, I think that was what made the difference. That contact, I wasn’t just another one, I didn’t feel like that, you know?* (P9 - 26 years old, mixed race, single, incomplete elementary school)

*She told me that she “admired me as a mother, that I would be a great mother”, you know? And that makes all the difference to me* [...] *but those things were what touched me the most, you know?* [...] *sorry, guys* [expresses emotion and tears]. (P7 - 22 years old, mixed race, married, complete high school)

Specifically, regarding breastfeeding, the available presence, with efforts and intentions for practical success, symbolized support and promoted differentiated and memorable support.

*She spent about 15 to 20 minutes there with me, helping me with [child’s name], and it was a blessing* [...] *it was a blessing for me*. [...] *she will stay in my memory forever. I think the day I have another child, I’ll immediately remember her, the breastfeeding thing. That she was very important* [...] *like* [...] *her being there* [...] *to have time like that.* (P3 – 36 years old, black, married, complete elementary school)

[...] *she came in like this, leaned on me, grabbed my shoulder, told me I could do it* [...] *and only left when she* [child] *was already breastfeeding* [...] *she leaned on me, said, come, understand? She didn’t just give me instructions and leave*. (P11 – 34 years old, mixed race, married, incomplete higher education)

The counselor’s affection and loving presence were highlighted, including by a participant who experienced postpartum complications and breastfed her child only a few times.

[...] *opened me up again because I wouldn’t stop bleeding*. [...] *I was in a coma for six days, and then I stopped breastfeeding, only at the very beginning*. [...] *the affection she [counselor] had* [...] *the attention she gave me* [...] *at the time we were* [...] *she welcomed me* [...] *me* [...] *with kind words* [...] *I cried a lot and she sat next to me, she talked, she held* [child’s name]*, so she helped me a lot* [...] *she’s a wonderful person*. (P2 – 21 years old, white, single, completed higher education)

### Counseling and decision-making

The counselor and her behavior integrated the mental conversations the woman had when faced with limits, difficulties, uncertainties, and/or insecurity in aspects related to breastfeeding or peripheral to it, such as pacifier use and nipple-areolar complex injuries. In the self-talk, between the “I” and the “me”, the counselor’s behavior helped mitigate the force of social impositions and promote self-emergence, guiding decision-making.

*The only person who encouraged me to breastfeed was* [counselor’s name]*,* [...] *if it weren’t for her, I might not have been able to, because she taught me everything back in the hospital, how* [child’s name] *should latch on and everything. I remembered her always saying she was in trouble and things would* [...] *go*. (P1 – 21 years old, mixed race, married, complete high school)

[...] *I said, “I couldn’t take it, woman, I gave the girl a pacifier, but she didn’t want it”. She said, “No, but you’ll see that* [...] *the way she’ll get used to it without the pacifier, she won’t need it. She doesn’t need a pacifier. She’ll be comforted by your breast”. That’s it, I picked up the pacifier and thought about it. And so it was. She didn’t even need the pacifier. The girl is still there, look, without a pacifier*. (P3 – 36 years old, black, married, complete high school)

Despite the above, some women faced difficulties in making respectable decisions regarding breastfeeding and the non-introduction of pacifiers, especially those who depended on social support for childcare due to the need to work. In this context, it was within the family that the main stakeholders met, feeling alone in not using pacifiers and bottles.

*Oh, to tell you the truth, I think I was alone, because everyone wanted me to bottle-feed my son, everyone, without exception. My mother, his father, everyone. The only person who encouraged me to breastfeed was [counselor’s name], but otherwise, no one wanted me to breastfeed. Everyone wanted me to wean him off the breast and bottle-feed him* [...] *my paternal grandmother tried so hard to introduce him to a bottle* [...] *but anyway, I didn’t say anything, because since we need help to work, I couldn’t say, “You’re not going to give my son a bottle”! But I didn’t like it. And she also tried to introduce him to a pacifier. But I never liked it, I always asked her, but even when I asked her not to, she always did, you know?* (P1 – 21 years old, mixed race, married, complete high school)

Furthermore, the counselor’s verbalization of faith in women, in their ability to choose in the breastfeeding process, supported decisions and investments.

[...] *when I had those difficult moments, like, my breasts were bleeding, it hurt a lot, I cried a lot, and I said, “No, I’m going to insist”* [...] *and I remember her saying, look, it’s your choice. But know how important that was* [...] *this more humane treatment, which wasn’t a rule, an obligation, that got me. That’s what held me together. I said, “Okay, that’s it, fine, done”* [...] *my decision to go ahead with breastfeeding came from a dialogue, something that had a big impact on me*. (P9 – 26 years old, mixed race, single, incomplete elementary school)

[...] *when she said it was possible, “it’s not impossible, you can do it”, that touched me, it encouraged me to really try, it really encouraged me*. (P11 – 34 years old, mixed race, married, incomplete higher education)

### Counseling symbolisms

Counseling provided support to the primiparous woman in the process of becoming a lactating mother, countering the anchoring in the meaning of being a “newbie” in the subject, with contributions to her empowerment for the experience of breastfeeding.

*I really liked the counseling* [...] *just knowing that you’re not alone there, that there’s someone there to try to help you, to do something for you. You don’t know anything, it’s all new to you, because we weren’t born knowing how to be mothers. So, like* [...] *it’s a really wonderful thing*. (P2 – 21 years old, mixed race, married, complete high school)

They felt equipped with emotional elements, knowledge and practical tips for managing breastfeeding, which reflected on the meaning of counseling as a rightful intervention for all women and a differentiated intervention to achieve breastfeeding.

[...] *All that care, that affectionate explanation, that patience makes a huge difference. So, for me, it was very important* [...] *those who don’t have it, I say they must miss it, because I say that if I had another pregnancy and didn’t have this, even though I’m already a mother, I think I would miss that support to face breastfeeding situations*. (P4 - 26 years old, mixed race, married, complete high school)

*Maybe, if she hadn’t been there, breastfeeding wouldn’t have been as good* [...] *she gave me a lot of advice, she taught me a lot*. (P7 - 22 years old, mixed race, married, complete high school)

*I think all postpartum women should receive this guidance on breastfeeding* [...] *women in the postpartum period need guidance* [...] *that postpartum counseling moment is essential. I was in a room with four postpartum women* [...] *this counselor, she didn’t do it for all four women in the room, no. And I saw that, despite my inverted nipple problem, I was the only postpartum woman who was able to breastfeed well* [...] *if it were possible, every hospital should have a person specialized solely in breastfeeding to provide more ongoing guidance to all postpartum women*. (P11 – 34 years old, mixed race, married, incomplete higher education)

## DISCUSSION

As understood from the statements of the study participants, the relevance of breastfeeding counseling provided by a nurse in the immediate postpartum period is evident, in the context of RI, as a contribution to decision-making regarding exclusive breastfeeding. Thus, counseling was recognized, valued, and endorsed as a strategy that promotes breastfeeding and supports women in the process of becoming lactating.

SI emphasizes the importance of human behavior and individual experiences, highlighting how people attribute, interpret, and transform the meanings of these experiences in their social context^(12)^.

In all the women interviewed, the meaning of counseling was linked to differentiated support, providing emotional elements, knowledge, and practical tips for managing breastfeeding. The interaction with the counselor reverberated in women’s inner world, allowing them to interact with themselves, interpreting and assigning meaning to their experiences, and consequently, planning and making decisions based on these interpretations, as indicated by a phenomenological study in which women, when they felt heard, seen, and welcomed, felt empowered to continue breastfeeding and overcome adversity^(10)^.

In their speeches, women report pillars of breastfeeding counseling, highlighting the counselor’s ability to listen, express empathy, welcome and validate feelings, and demonstrate interest and connection with the uniqueness of each woman’s experience, paying attention to non-judgment, as well as focusing on the development of relational trust^(5,18)^. For these outcomes, the indications of counseling are acceptance of a person’s perspective, focusing on positive aspects rather than mistakes, including offering praise^(5,18)^. Furthermore, offering practical help and useful, timely information, using clear, simple language and offering few suggestions, is listed in the indications of breastfeeding counselor training^(5,18)^, being highlighted by participants and reinforcing the relevance of these guidelines.

Person-centered breastfeeding counseling responds to the needs, preferences, and values of the families being assisted. A systematic review of qualitative studies on mothers’ values and preferences showed that women desire autonomy and for their choices to be respected, and those who did not breastfeed or were unable to breastfeed felt unsupported and unheard^(4)^. In postpartum women’s statements, it is clear that the counselor places the woman at the center and focus of breastfeeding, and they feel welcomed by the approach.

In addition, counseling also involves the clinical management of breastfeeding, which includes observation and evaluation of breastfeeding, which may include assistance in positioning the mother and newborn, in addition to the clinical management of common breast complications during this period, such as sore nipples, injured nipples, congestion, mastitis, and apparently insufficient milk^(19)^. It is important to emphasize that this management should be guided by postpartum women’s need for support and if requested by them. However, it is emphasized that counseling in cases where breastfeeding problems are detected increases the chances of maintaining exclusive breastfeeding^(20)^. At this point, it is noted that some women sought support from the counselor, identifying the relevance of her help in managing breastfeeding, contributing to solving their doubts and concerns.

Evidence indicates that the use of breastfeeding counseling in RI enabled the successful practice of breastfeeding, even in unfavorable or difficult clinical management cases^(21)^, increasing the chances of maintaining it exclusively until four^(8,22)^ and six months^(9,23)^ and reducing the chances of using formulas during hospitalization in RI^(24)^. Furthermore, studies indicate that empowering nursing mothers through counseling reduced the belief of insufficient or weak milk production, the main cause of weaning^(25,26)^. The results achieved here advance the evidence and signal the relevance of the process of becoming a nursing mother for primiparous women.

However, even in light of the evidence, and despite being a strategy described and highly recommended by the WHO, the practice is uncommon, considering that more than half of the world’s population does not have full access to essential healthcare services, including access to counseling by trained professionals^(27)^, which negatively impacts the support received by women during breastfeeding.

It is noteworthy that half of lactating women have difficulty breastfeeding in the first three days after birth, requiring support^(28)^. Furthermore, a study found that primiparous women have more difficulty initiating breastfeeding than multiparous women, delaying its initiation^(29)^, which can compromise breastfeeding success. The results obtained here indicate the need for breastfeeding counseling in RI as a counterpoint. This is mainly due to the relational reach achieved, in which the person feels comfortable and welcomed in the presence offered by a counselor. A counselor’s behavior is essential to the act of counseling.

In contrast, family insistence on the use of nipples and bottles, especially when women depend on their family network for support in childcare when they go to work, puts exclusive breastfeeding at risk, as emerged in the statements, regardless of advice.

The studies presented point to the importance of support for primiparous women and the effectiveness of the breastfeeding counseling strategy to overcome difficulties and maintain exclusivity as recommended.

The possibility of weaning arises in the face of complications and difficulties. One participant who had a postpartum complication and found that counseling was insufficient to maintain breastfeeding is noteworthy. An institutional philosophy was needed that valued and anticipated the need to maintain the woman as a lactating mother, and then consider relactation.

The evidence presented here suggests that RI is a distinct space for counseling, as it is articulated with a specific moment in the immediate postpartum period, with evidence of qualitative exploration in this area. Furthermore, although the scope of this study was not explored, the counselors were registered nurses, all with counseling training, which suggests the existence of a specific competency profile. Therefore, we suggest the development of studies focusing on the counselor’s interactional competencies, given the emphasis the participants placed on this area. In any case, nurses are suggested as professionals with characteristics that favor breastfeeding counseling.

Furthermore, the experiences experienced in counseling interactions reverberate in women’s memories, being revived in situations related to breastfeeding in internalized conversations, guiding decision-making. This longitudinal aspect of counseling is a relevant finding and an advancement in existing evidence, provided by the present study.

### Study limitations

This study has limitations, particularly related to reporting the experiences of individuals exposed to the breastfeeding counseling intervention developed by three nurses. Therefore, studies exploring the scope of counseling provided by other professional categories and in other care settings could inform the findings of this investigation.

The remote interviews can be considered a weakness of the study; however, the interviewers’ use of cameras and the investment in creating a welcoming relational context sought to promote narrative and data density. The importance of developing new studies with in-person interviews is reinforced; however, it is emphasized that the interviews’ remote nature facilitated the availability of participants.

Another limitation is that three women who participated in the pilot study were excluded because they did not have a smartphone, internet access, or both. This limitation compromises the generalizability of results, as it does not encompass situations of social vulnerability expressed in the diversity of access to technology. To address this gap, we emphasize the importance of developing new studies that interview women in their homes or in controlled settings.

Furthermore, the study was conducted only with primiparous women and in three hospitals, with a small number of participants, which compromises the generalizability and transferability of results. However, qualitative studies do not have this intention.

### Contributions to nursing, health, or public policy

The results obtained advance and strengthen the evidence on breastfeeding counseling, especially regarding professional presence as a key element in the constructive relationship of the intervention and the impact of this relationship on breastfeeding decision-making and its related aspects. The reports reinforce the importance of incorporating breastfeeding counseling into professional training, staffing, and public policies to promote and protect breastfeeding. Furthermore, the study emphasizes the importance of nurses as highly relevant agents in counseling actions, as evidenced in the study.

## FINAL CONSIDERATIONS

Women pointed out elements of the essential pillars of breastfeeding counseling and their influence on the decision-making process to maintain exclusive breastfeeding in the first year of their children’s lives, highlighting the effects of the behavior of the presence of counseling nurses on care relationship and its scope.

## Data Availability

The research data are available within the article.
