# Supplementary material for: Decision-making about breastfeeding: influences of breastfeeding counseling
Source: Rev Bras Enferm. 2026 Jan 9;78(Suppl 3):e20240599. doi: 10.1590/0034-7167-2024-0599 (PMC12798866; doi:10.1590/0034-7167-2024-0599)
Supplement: Translation

# Translation: Tomada de decisão acerca da amamentação: influências do aconselhamento em aleitamento materno

**DOI:** 10.1590/0034-7167-2024-0599pt

**Authors:** Jéssica Aparecida da Silva, Monika Wernet, Allison Scholler de Castro Villas Boas, Elisa da Conceição Rodrigues, Karine Emanuelle Peixoto Oliveira da Silva, Mariana Torreglosa Ruiz

**Affiliations:** I Universidade Federal do Triângulo Mineiro. Uberaba, Minas Gerais, Brasil.; II Universidade Federal de São Carlos. São Carlos, São Paulo, Brasil.; III Universidade Federal do Rio de Janeiro. Rio de Janeiro, Rio de Janeiro, Brasil.; IV Universidade Estadual de Feira de Santana. Feira de Santana, Bahia, Brasil.

**Keywords:** Aconselhamento, Tomada de Decisões, Aleitamento Materno, Desmame, Pesquisa Qualitativa

## Abstract

**Objetivos:**

compreender como mulheres que receberam aconselhamento em aleitamento materno tomam decisões relacionadas à amamentação durante o primeiro ano de vida de suas crianças.

**Métodos:**

estudo qualitativo, realizado junto a mulheres que receberam aconselhamento em aleitamento materno em unidades de Alojamento Conjunto de três hospitais universitários, dois localizados na região Sudeste e um na região Nordeste. Realizaram-se entrevistas semiestruturadas, entre maio e setembro de 2024, apreciadas sob os referenciais do Interacionismo Simbólico e da Análise Temática.

**Resultados:**

participaram 11 mulheres, que destacaram o contexto interacional do aconselhamento em aleitamento materno como promotor de segurança e confiança, com contribuições técnicas e emocionais para o enfrentamento de desafios na amamentação.

**Considerações Finais:**

o aconselhamento em aleitamento materno se revelou como tecnologia de cuidado com influências positivas para a manutenção do aleitamento materno exclusivo no primeiro ano de vida de crianças.

## INTRODUÇÃO

Apesar de as evidências endossarem a recomendação para a amamentação e os benefícios do leite humano^(1)^, menos da metade das crianças é amamentada exclusivamente até o sexto mês de vida^(2)^, índice semelhante à prevalência do aleitamento humano exclusivo até os seis meses de idade no Brasil^(3)^. Esses dados evidenciam elevadas e alarmantes taxas de desmame mundial, assim como nas crianças brasileiras^(4,5)^.

A Organização Mundial da Saúde (OMS) indica e reconhece o aconselhamento em amamentação como tecnologia para prevenção e promoção do aleitamento humano^(4)^. Estudo de revisão sistemática com metanálise sustentou o aconselhamento enquanto intervenção eficaz em saúde pública para o aumento das taxas de duração da amamentação, inclusive a exclusiva^(6)^. O aconselhamento em aleitamento humano transcende o manejo clínico e orientações, estruturando-se na relação horizontal e dialógica entre profissional (conselheiro) e pessoa que amamenta ou pretende amamentar, baseado na Terapia Centrada no Cliente de Carl Rogers^(7)^. Sua proposta é de apoiar a amamentação, com consideração e respeito às particularidades e desejos das mulheres que amamentam ou intencionam amamentar^(4,5)^.

O profissional não é responsável por determinar o que e como uma mulher deve fazer, mas deve prover o suporte para que ela tome decisões com consideração aos aspectos singulares de sua vivência^(5)^. Para tanto, a indicativa é de que o profissional realize a capacitação em aconselhamento em amamentação, integrando aspectos teóricos e práticos. Essa capacitação intenciona desenvolver no aconselhador habilidades complexas de comunicação fundamentadas nos conhecimentos e habilidades para o suporte na amamentação, com alinhamento ao projeto, realidade e possibilidades de cada mulher e família^(4,5,8)^. Tem como pilar a centralidade da relação entre o profissional e a pessoa aconselhada, com investimentos na habilidade da escuta, valorização da empatia e elogios, e desencorajamento do julgamento, com vistas ao aumento da confiança da mulher e família para o ato de amamentar^(4,5,8)^.

Ensaio clínico randomizado realizado com 102 primíparas, no qual foram aplicadas sessões de aconselhamento sobre aleitamento materno humano durante a internação da díade no Alojamento Conjunto (AC), em comparação aos cuidados habituais, indicou que as mulheres que receberam a intervenção tiveram maior probabilidade de manter o aleitamento materno exclusivo até o sexto mês de vida^(9)^, demonstrando alcance da estratégia a longo prazo.

Ainda nesse sentido, estudo qualitativo com abordagem fenomenológica realizado na Noruega apontou que as lactantes estavam refletindo sobre a possibilidade do desmame quando receberam suporte através do aconselhamento, e o mesmo foi essencial para que não o interrompesse, que se (re)estabelecesse e continuassem a amamentar. As mesmas enfatizaram que a abordagem fez com que fossem percebidas tanto como mães que amamentam quanto como mulheres com necessidades próprias^(10)^.

As evidências apontadas, de natureza quantitativa e qualitativa, demonstram o impacto do aconselhamento em aleitamento realizado por profissional capacitado na tomada de decisão das mulheres que intencionam amamentar.

Diante dos altos índices de desmame precoce, das possíveis repercussões para a saúde da criança e da necessidade de adensar conhecimentos sobre aconselhamento em aleitamento materno, devido à escassez de estudos sobre a temática, mesmo diante das evidências de sua eficácia, o fenômeno posto em exploração foi a compreensão do impacto do aconselhamento em aleitamento materno na tomada de decisão em amamentação que justifica sua realização.

## OBJETIVOS

Compreender como mulheres que receberam aconselhamento em aleitamento materno tomam decisões relacionadas à amamentação durante o primeiro ano de vida de suas crianças.

## MÉTODOS

### Aspecto éticos

O estudo obteve aprovação ética pelo Comitê de Ética em Pesquisa, sob Parecer nº 6.274.311, de 31 de agosto de 2023 (Certificado de Apresentação para Apreciação Ética nº 4 61321122.3.1001.8667), utilizando as diretrizes e normas regulamentadoras de pesquisas envolvendo seres humanos contidas na Resolução Nacional nº 510/2012/CNS/MS, Também cumpriu os princípios éticos contidos na Declaração de Helsinki, sendo assegurado que o Termo de Consentimento Livre e Esclarecido fosse assinado por todas as participantes do estudo.

### Tipo de estudo

Trata-se de pesquisa qualitativa, cujo desenvolvimento e relatório consideraram o *COnsolidated criteria REporting Qualitative research* (COREQ)^(11)^.

### Referencial teórico-metodológico

Este estudo adotou como referencial teórico-metodológico o Interacionismo Simbólico (IS), para o qual as pessoas se comportam a partir das interpretações e significados estabelecidos nos processos interacionais, tanto consigo quanto com o(s) outro(s)^(12)^. O sentido, o significado e o comportamento estão sempre abertos, passíveis à transformação, dependentes do que emerge da interação social^(12)^.

O ato de amamentar está sob a influência dos sentidos e significados que a pessoa tem sobre si, de seu corpo, da alimentação, do papel materno, da amamentação, do amamentar, e da relevância deles para ela, sua família e sociedade. Portanto, o aleitamento é uma prática social complexa que envolve a auto-interação e, concomitantemente, as interações sociais ampliadas^(13)^.

### Procedimentos metodológicos

As participantes deste estudo integravam o grupo intervenção de estudo piloto de ensaio clínico randomizado (ECR), desenvolvido em três centros, voltado a investigar a eficácia do aconselhamento em aleitamento materno durante a internação da díade (pessoa da mãe e seu recém-nascido) no AC, seu impacto na duração e manutenção do aleitamento exclusivo^(14)^. O grupo intervenção recebeu, durante a internação no AC, de duas a quatro sessões de aconselhamento em aleitamento materno. Cada centro contou com uma aconselhadora, enfermeira de formação, capacitada em aconselhamento em aleitamento materno (formação teórico-prática de 76 horas). A escolha por profissional enfermeiro se deveu ao fato de a literatura apontar ser o profissional mais citado pelas mulheres nas ações de proteção e promoção do aleitamento materno^(15)^. Ressalta-se que, para evitar possíveis vieses de resposta, formaram-se equipes distintas para os diferentes objetivos dos estudos. Foram designadas pesquisadoras que aplicaram a intervenção do estudo piloto de ECR, e as pesquisadoras que realizaram as entrevistas que compõem este estudo não participaram da coleta do estudo de intervenção.

Os critérios para a participação no estudo piloto de ECR foram primíparas, com criança filha única, nascida viva, idade gestacional de 37 a 42 semanas (termo) e peso superior a 2.500 gramas, independentemente do tipo de parto. Os critérios de não inclusão foram: puérperas e neonatos com contraindicação para o aleitamento; neonatos com malformações que impedissem ou dificultassem o aleitamento e/ou com alteração da mecânica do aleitamento (frênulo lingual); puérperas cujos neonatos fossem imediatamente separados após o clampeamento do cordão umbilical ao nascimento, devido a intercorrências materno-neonatais; puérperas transferidas de outras instituições ou que já tinham recebido alta (reinternação) na alocação; puérperas usuárias de drogas ilícitas e etilistas; e puérperas com deficiências intelectual e/ou sensoriais, a partir de diagnóstico registrado em prontuário.

Para o presente estudo, consideraram-se todas as participantes do grupo intervenção do estudo piloto que referiram ter acesso à internet, condições e recursos para realizar videochamadas. O convite para integrá-lo ocorreu no contexto das ligações de seguimento do estudo piloto do ECR, com reforço via mensagem escrita via *WhatsApp*®. Na ocorrência de manifestação de interesse de participação, era enviado Termo de Consentimento Livre e Esclarecido (*online*), sendo realizado agendamento da entrevista em dia e horário de preferência da participante.

As entrevistas ocorreram no ambiente *online*, por meio de videochamadas na plataforma *WhatsApp*®, com gravação de áudio mediada por um aparelho gravador. As entrevistas foram conduzidas por duas pesquisadoras: primeira e terceira autoras deste manuscrito. Ambas receberam, previamente à coleta dos dados, formação em entrevistas qualitativas e nos referenciais do estudo (somaram 60 horas) ministrado pela segunda autora, pesquisadora com *expertise* no desenvolvimento de estudos qualitativos. Ademais, a coleta e a análise de dados ocorreram de forma concomitante, sempre sob apoio e supervisão da terceira e última autora, aspecto que favoreceu dados densos.

O uso ou não da câmera pela participante ficou respeitado, porém ambas as entrevistadoras mantiveram suas câmeras ligadas, sob intenção de que as comunicações faciais e gestuais, assim como outros elementos da paralinguagem, contribuíssem com o estabelecimento de um conforto interacional na coleta dos dados e comunicassem o interesse pelo narrado. No início da videochamada, foi solicitada à participante autorização para gravação da mesma, sendo explicada de forma clara sua finalidade.

Para contrapor as limitações da coleta por videochamada, listamos os cuidados procedimentais adotados. Para minimizar a diversidade de acesso às tecnologias, as mulheres tinham a opção de manter a câmera aberta ou não durante as entrevistas, e todas foram audiogravadas, de forma que a qualidade da tecnologia não interferiu nos resultados apresentados. Já o fato de as entrevistas não serem realizadas em ambiente controlado, onde não é possível controlar ruídos no ambiente e nas falas, e distrações, podendo haver interrupção da participante, adotou-se como critério o agendamento das entrevistas em horário oportuno para as participantes. Os pesquisadores foram calibrados com habilidades de comunicação para voltar ao ponto de dúvida ou retomar após interrupções. Quanto à limitação da não captação da linguagem não verbal, ressalta-se que a audiogravação permitiu identificar pausas e emoções nas falas nos casos em que a participante permaneceu com a câmera desligada. Desta forma, foram tomados cuidados para superar ou minimizar as limitações da estratégia adotada.

### Cenário do estudo

O estudo piloto do ECR foi realizado em três centros, nas respectivas unidades de AC. Tratam-se de hospitais universitários, com atendimento público, localizados em Minas Gerais, Rio de Janeiro e Bahia.

Na instituição situada em Minas Gerais, a unidade em que foram alocadas as mulheres é constituída por 12 enfermarias de AC individualizadas, onde permanecem juntos a díade e seu acompanhante. A instituição não possui título de Hospital Amigo da Criança, embora esteja em suas metas. É referência para resolução de gestações de alto risco e de gestações normais de sua área de abrangência, além de assistir 27 municípios, incluindo aqueles que não possuem maternidade. A instituição possui protocolo operacional padrão para assistência à amamentação, entretanto os procedimentos contemplam apenas ações de manejo clínico do aleitamento.

O hospital do Rio de Janeiro oferece assistência ambulatorial, hospitalar e multiprofissional na atenção à saúde de gestantes e recém-nascidos de alto risco. A unidade de AC possui nove enfermarias, compostas por cinco leitos cada, totalizando 45 leitos, e a instituição recebeu o título de Hospital Amigo da Criança em dezembro de 2020.

O hospital da Bahia e é credenciado como Hospital Amigo da Criança desde 1995. Referência para resolução de gestações de baixo risco, a instituição pública de ensino possui três unidades de AC com capacidade instalada de atendimento a 95 leitos.

### Amostra

Um total de 29 participantes foram as participantes em potencial (grupo intervenção do estudo piloto ECR) para a presente pesquisa. Contudo, três mulheres não foram incluídas por não terem condições e recursos para receber ligações por videochamada, 12, por não responderem após três tentativas de contato das pesquisadoras, e três, por recusarem a participar, sob a justificativa de não dispor de tempo para se envolver com a entrevista.

Na nona entrevista, não surgiram novos elementos de composição dos temas, havendo indícios de densidade de dados. Porém, como restavam duas mulheres para abarcar a totalidade das participantes que sinalizaram interesse e disponibilidade de participação, as entrevistas com elas foram realizadas e evidenciaram a saturação de dados^(16)^.

### Coleta e organização dos dados

A coleta de dados ocorreu entre maio e setembro de 2024. As entrevistas foram audiogravadas, com duração média de 30 minutos, utilizando-se roteiro semiestruturado. O roteiro contou com as seguintes questões: como vem alimentando a sua criança desde o nascimento até os dias atuais? Relembre os contatos com a aconselhadora em aleitamento materno. O que foi significante pra você? Como esses contatos influenciaram a sua tomada de decisão em relação à amamentação? Outras perguntas alinhadas ao exposto foram apresentadas sempre que era necessário ampliar a compreensão do narrado.

As entrevistas foram transcritas com o auxílio do aplicativo Transkriptor® e transferidas para documento Word® pela primeira autora, sendo conferidas posteriormente na íntegra pela segunda autora. Para preservar a identidade das participantes, estas foram identificadas por ordem numérica de realização das entrevistas (*e.g*., P1, P2...), e os nomes citados durantes as falas foram substituídos pela letra inicial. Ressalta-se que as transcrições, após a conferência pelas pesquisadoras, foram enviadas através do *WhatsApp*® para as participantes, com solicitação de ajustes na necessidade, o que não ocorreu, sendo, assim, consideradas validadas.

### Análise dos dados

Os documentos de áudio transcrição foram submetidos ao processo de análise temática, proposto por Braun e Clarke (2006), utilizando o *software* Atlas.ti® para o agrupamento de informações. O processo analítico, desenvolvido pela primeira, segunda e terceira e última autoras, percorreu seis fases: familiarização com os dados; geração códigos iniciais; busca de temas; revisão de temas; definição e nomeação de temas; e produção do relatório^(17)^. Os demais autores integraram reuniões de discussão da análise, trazendo contribuições.

Ressalta-se que, durante o processo de análise, foram realizadas leituras repetidas das transcrições de forma ativa, à procura de significados, inclusive das outras informações latentes das experiências das participantes, na busca do significado subjacente das palavras. Concomitante a isso, foram gerados códigos iniciais a partir das narrativas transcritas, os quais identificaram características comuns dos dados, dando origem aos temas (unidade de análise principal e mais ampla)^(17)^.

## RESULTADOS

### Caracterização das participantes

Participaram do estudo 11 mulheres, sendo quatro assistidas no hospital de Minas Gerais. Quatro, no hospital do Rio de Janeiro e três, no hospital da Bahia. As mulheres tinham idade média de 27, variando de 21 a 35 anos. Oito mulheres relataram viver com seu companheiro, e três se declararam solteiras. Oito mulheres se autodeclararam pardas, todas alfabetizadas, e uma possuía ensino fundamental completo, duas, ensino médio incompleto, seis, ensino médio completo, uma, ensino superior incompleto, e uma havia concluído o curso de ensino superior. Seis mulheres exerciam atividades laborais remuneradas, e a renda familiar variou entre um e três salários mínimos.

Com relação à via de nascimento, cinco tiveram parto vaginal, enquanto seis foram submetidas à cesárea. A idade das crianças no período da coleta de dados variou de 14 a 18 meses, com média de 15 meses. Todos receberam aleitamento materno em algum momento da vida, sendo que a maioria (n=7) permaneceu em amamentação exclusiva por seis meses, e delas, seis ainda mantinham o aleitamento materno na data da entrevista.

A análise possibilitou a identificação de três temas: “Comportamento de presença e desdobramentos de cuidado”; “Aconselhamento e tomada de decisão”; e “Simbolismos do aconselhamento” (Quadro 1).

**Quadro 1:** Temas, subtemas e códigos do estudo “Tomada de decisão acerca da amamentação: influências do aconselhamento em aleitamento materno”, Uberaba, Minas Gerais, Brasil, 2024

Códigos	Subtema	Tema
Respeito e disponibilidade	Presença implicada	Comportamento de presença e desdobramentos de cuidado
Escuta carinhosa		
Mutualidade		
Consideração ao tornar-se mãe	Acolhimento	
Consideração às possibilidades de amamentação		
Intercorrências na amamentação	Intervenientes na amamentação	Aconselhamento e tomada de decisão
Bicos, mamadeiras e manutenção da amamentação		
Imposições sociais e amamentação		
Incentivos	Suporte da aconselhadora	
Memórias do aconselhamento		
Ser nutriz e primípara	Reconhecimento	Simbolismos do aconselhamento
Primípara e maternidade		
Dicas técnicas	Parceria	
Suporte emocional		

O aconselhamento em aleitamento materno emerge nas falas como um apoio técnico e emocional às mães, ajudando-as a tomar decisões e a enfrentar os desafios da amamentação com mais segurança e confiança. Apesar disso, em algumas situações, não foi suficiente para fazer contraponto aos intervenientes, com destaque às crenças e valores familiares.

### Comportamento de presença e desdobramentos de cuidado

As mulheres assinalaram o comportamento de presença da enfermeira aconselhadora como diferencial para a experiência, de modo a ser significado como de reconhecimento, interesse e respeito à sua pessoa e ao processo de estarem tornando-se mães. O comportamento de presença esteve permeado por afetividade, favorecendo um processo interacional de mutualidade e acolhimento, com conforto relacional. Mulheres se sentiram percebidas, ouvidas, valorizadas e apoiadas em questões da amamentação, mas também do tornar-se mãe e nas questões emocionais.

[...] *eu me senti muito acolhida por ela* [...] *parecia que a gente se conhecia há milhões de anos já* [...] *o carinho que ela teve, a atenção que ela me deu* [...] *porque eu já tinha passado por um momento muito difícil que foi o meu parto* [...] *então, ela me acolheu com palavras de carinho* [...] *eu chorava muito* [...] *porque eu estava depressiva* [...] *então ela sentava do meu lado* [...] *ela conversava* [...] *ela pegava a* [nome da criança]*, então ela me ajudou demais*. (P2 – 21 anos, parda casada, ensino médio completo)

*Quando ela sentou e conversou comigo, e ela me explicou, foi um bate-papo. Ela não chegou para mim e falou assim: “Olha, você tem que fazer isso, porque isso, aquilo, outro”. Acho que foi a maneira como ela conduziu a conversa, porque a gente, principalmente quando você está ali e quando você, mãe de primeira viagem, eu precisava desse acolhimento em fala mesmo, eu precisava que olhassem no meu olho, acho que isso foi o diferencial. Esse contato, eu não era só mais uma, eu não me sentia assim, sabe?* (P9 – 26 anos, parda, solteira, ensino fundamental incompleto)

*Ela falou comigo que “me admirou como mãe, que eu seria uma ótima mãe”, sabe? E isso faz toda a diferença pra mim* [...] *mais essas coisas foram o que mais me tocou, sabe?* [...] *desculpa, gente* [expressa emoção e choro]. (P7 - 22 anos, parda, casada, ensino médio completo)

Especificamente sobre a amamentação, a presença disponível, com esforços e intenções de sucesso prático, simbolizou suporte e promoveu um suporte diferenciado e memorável.

*Passava ali uns 15 a 20 minutos lá comigo, me ajudando com* [nome da criança]*, e era uma benção* [...] *foi uma benção para mim*. [...] *ela vai ficar na minha lembrança para sempre assim. Acho que no dia em que eu tiver outro filho, eu vou logo lembrar dela, a questão da amamentação. Que ela foi muito importante* [...] *assim* [...] *dela estar presente* [...] *ter um tempo assim*. (P3 – 36 anos, negra, casada, ensino fundamental completo)

[...] *ela chegou assim, encostou em mim, pegou no meu ombro, falou que eu conseguia* [...] *e só saiu quando ela* [criança] *já estava realmente mamando* [...] *encostou, falou, chegou, entendeu? Não só orientou e tchau*. (P11 – 34 anos, parda, casada, ensino superior incompleto)

O carinho e a presença afetiva da aconselhadora estiveram ressaltados, inclusive por participante que vivenciou intercorrência pós-parto e amamentou sua criança por poucas vezes.

[...] *me abriu de novo porque eu não parava o sangramento*. [...] *eu fiquei em coma seis dias, daí eu não amamentei mais, só bem no iniciozinho*. [...] *o carinho que ela [aconselhadora] teve* [...] *a atenção que ela me deu* [...] *no momento em que a gente estava* [...] *ela me acolheu* [...] *me* [...] *com palavras de carinho* [...] *eu chorava muito e ela sentava do meu lado, ela conversava, ela pegava a* [nome da criança]*, então ela me ajudou demais* [...] *ela é uma pessoa maravilhosa*. (P2 – 20 anos, branca, solteira, ensino superior completo)

### Aconselhamento e tomada de decisão

A aconselhadora e seu comportamento integraram as conversas mentais que a mulher tecia diante de limites, dificuldades, incertezas e/ou insegurança em aspectos relativos à amamentação ou periféricos a ela, a exemplo do uso de chupetas e lesões no complexo mamilo-areolar. Na conversa no *self*, entre o “eu” e o “mim”, o comportamento da conselheira contribuía para atenuar a força das imposições sociais e promover o emergir de si, direcionando a tomada de decisão.

*A única pessoa que me incentivava a dar mamá era só a* [nome da aconselhadora], [...] *se não fosse ela, talvez eu não conseguiria, porque ela que me ensinou tudo lá no hospital, como que tinha que ser a pegada do* [nome da criança] *e tudo mais. Lembrava dela falando sempre que tinha perrengue e a coisa ia* [...] *foi*. (P1 – 21 anos, parda, casada, ensino médio completo)

[...] *eu falava assim: “Eu não aguentei não mulher, taquei chupeta na menina, mas a menina não quis não”. Ela fez: “Não, mas aí você vai ver que* [...] *do jeito que ela vai se acostumar sem a chupeta, não vai precisar. Ela não precisa de chupeta. Ela vai se acalentar no seu peito”. Pronto, eu pegava na chupeta e pensava nisto. E assim foi. Nem precisou da chupeta, não. A menina tá aí, ó, até hoje sem chupeta*. (P3 – 36 anos, negra, casada, ensino fundamental completo)

Apesar do exposto, algumas enfrentaram dificuldades em tomar decisões relativas à amamentação e à não introdução de bicos respeitadas, sobretudo aquelas que eram dependentes do suporte social para o cuidado da criança diante da necessidade de trabalhar. Nesse contexto, foi no âmbito da família que se encontraram os principais intervenientes, sentindo-se solitárias no não uso de bicos e mamadeiras.

*Ah, pra te falar a verdade, eu acho que eu estava sozinha, porque todo mundo queria que eu chuchasse mamadeira no meu filho, todo mundo, sem exceção. Minha mãe, o pai dele, todo mundo. A única pessoa que me incentivava a dar mamá era só a* [nome da aconselhadora]*, mas de resto, ninguém queria que eu amamentasse. Todo mundo queria que eu tirasse o peito e desse mamadeira* [...] *a avó paterna tentava muito introduzir mamadeira nele* [...] *mas enfim, eu não falei nada, porque como a gente precisa de ajuda para trabalhar, eu não podia falar: “Você não vai dar uma mamadeira para o meu filho”! Mas eu não gostei. E ela também tentou introduzir chupeta nele. Só que eu nunca gostei, sempre pedia para ela, mas sempre mesmo eu pedindo pra não fazer, ela sempre fazia, sabe?* (P1 – 21 anos, parda, casada, ensino médio completo)

Ademais, a verbalização de credo da aconselhadora na mulher, na sua capacidade de escolha no processo da amamentação, sustentou decisões e investimentos.

[...] *quando eu tive esses momentos mais difíceis, assim, meu peito sangrou, doía muito, eu chorava muito, que eu falei: “Não, eu vou insistir”* [...] *e eu lembro muito ela falando, olha, é uma escolha sua. Mas saiba o quanto isso foi importante* [...] *esse tratamento mais humano, que não era uma questão de regra, uma obrigação, isso me pegou. Foi isso que me segurou. Eu falei: “Ok, é assim, beleza, pronto”* [...] *a minha decisão de ter ido à frente com a amamentação partiu por um diálogo, algo que me marcou muito*. (P9 – 26 anos, parda, solteira, ensino fundamental incompleto)

[...] *quando ela falou que era possível, “não é impossível, você pode conseguir”, isso mexeu comigo, isso me incentivou a tentar mesmo, me incentivou muito*. (P11 – 34 anos, parda, casada, ensino superior incompleto)

### Simbolismos do aconselhamento

O aconselhamento representou para a mulher primípara suporte no processo de tornar-se nutriz, contrapondo a ancoragem no significado de ser “novata” no assunto, com contribuições para seu empoderamento para a vivência da amamentação.

*Gostei bastante do aconselhamento* [...] *só de saber que você não tá sozinha ali, que tem uma pessoa pra tentar te ajudar, fazer alguma coisa por você ali. Você não sabe de nada, é tudo novo pra você, porque a gente não nasceu sabendo ser mãe. Então, tipo assim* [...] *é uma coisa muito maravilhosa*. (P2 – 21 anos, parda, casada, ensino médio completo)

Perceberam-se munidas de elementos emocionais, de conhecimentos e de dicas práticas para manejar a amamentação, o que reverberou no significado do aconselhamento como intervenção de direito a todas as mulheres e diferenciada para alcançar a amamentação.

[...] *faz muita diferença todo aquele cuidado, aquele carinho em explicar, aquela paciência. Então, pra mim, foi muito importante* [...] *quem não tem, eu falo que deve sentir falta, porque eu falo que se eu tivesse outra gestação e eu não tivesse isso, por mais que eu já sou mãe, eu acho que eu sentiria falta desse acolhimento para enfrentar situações da amamentação*. (P4 – 26 anos, parda, casada, ensino médio completo)

*Talvez, se ela não tivesse ido lá, não seria tão boa a amamentação* [...] *ela me deu muitos conselhos, ela me ensinou bastante*. (P7 - 22 anos, parda, casada, ensino médio completo)

*Eu acho que deveria ser algo que todas as puérperas deveriam receber, essa orientação sobre o aleitamento materno* [...] *a mulher no puerpério, a mulher precisa de orientação* [...] *aquele momento de aconselhamento pós-parto é essencial. Eu fiquei num quarto com quatro puérperas* [...] *essa aconselhadora, ela não fez em todas as quatro mulheres que estavam no quarto, não. E eu vi que, apesar do meu problema do mamilo invertido, eu era a única puérpera que estava conseguindo amamentar bem* [...] *se fosse possível, todo hospital deveria ter uma pessoa especializada só em aleitamento materno para estar orientando todas as puérperas de forma mais presente*. (P11 – 34 anos, parda, casada, ensino superior incompleto)

## DISCUSSÃO

Conforme compreendido nas falas das participantes do estudo, evidencia-se a relevância do aconselhamento em aleitamento materno desenvolvido por enfermeira no puerpério imediato, no contexto do AC, como de contribuição para tomada de decisão pela aposta no aleitamento materno exclusivo. Destarte, o aconselhamento foi reconhecido, valorizado e endossado como estratégia que favorece a prática da amamentação e acolhe a mulher no processo de tornar-se nutriz.

O IS enfatiza a importância do comportamento humano e das experiências individuais, destacando como as pessoas atribuem, interpretam e transformam os significados dessas vivências no seu contexto social^(12)^.

Em todas as falas das mulheres entrevistadas, o significado do aconselhamento esteve atrelado a um suporte diferenciado, munido de elementos emocionais, de conhecimentos e de dicas práticas para manejar a amamentação. A interação com a aconselhadora reverberou efeitos no mundo interno da mulher, permitindo-lhe interagir consigo mesmo, interpretando, atribuindo significados à experiência vivida e, consequentemente, planejando e tomando decisões com base nessas interpretações, tal como apontado por estudo fenomenológico em que as mulheres, ao se sentirem ouvidas, vistas e acolhidas, sentiram-se empoderadas em manter o aleitamento materno, superando as adversidades^(10)^.

As mulheres relatam em suas falas pilares do aconselhamento em aleitamento materno, com destaque para a habilidade do aconselhador em ouvir, manifestar empatia, acolher e validar sentimentos, e demonstrar interesse e conexão pela singularidade da experiência vivida por cada mulher, atentando-se para o não julgamento, assim como apostas no desenvolvimento de confiança relacional^(5,18)^. Para esses alcances, os indícios do aconselhamento são de aceitação da perspectiva da pessoa com foco nos aspectos positivos e não nos erros, inclusive realizando elogios^(5,18)^. Ainda, a oferta de ajuda prática e de informação útil e pontual, sob uso de linguagem clara e simples, com emissão de poucas sugestões, está arrolada nos indícios da formação de aconselhador em amamentação^(5,18)^, sendo destacada pelas participantes e reforçando a relevância desses balizadores.

O aconselhamento em aleitamento materno centrado nas pessoas responde às necessidades, preferências e valores das famílias assistidas. Revisão sistemática de estudos qualitativos sobre os valores e preferências das mães mostrou que as mulheres desejam autonomia e que suas escolhas sejam respeitadas, e aquelas que não amamentaram ou não conseguiam amamentar sentiam que não eram apoiadas ou ouvidas^(4)^. Nas falas das puérperas, fica nítido que o aconselhador coloca a mulher como centro e foco da amamentação, e as mesmas se sentem acolhidas com a abordagem.

Além disso, o aconselhamento também envolve o manejo clínico da amamentação, que inclui a observação e avaliação da amamentação, podendo se configurar como ajuda no posicionamento da mãe e do recém-nascido, além do manejo clínico de complicações mamárias comuns nesse período, como mamilos doloridos, mamilos feridos, congestão, mastite e leite aparentemente insuficiente^(19)^. Ressalta-se que esse manejo deve ser guiado pela necessidade de apoio das puérperas e se solicitado pelas mesmas. No entanto, enfatiza-se que o aconselhamento em casos onde se detectam problemas na amamentação aumenta as chances de manutenção do aleitamento materno exclusivo^(20)^. Neste ponto, nota-se que algumas mulheres recorreram ao suporte da aconselhadora, identificando a relevância do auxílio da mesma no manejo da mamada, contribuindo para sanar suas dúvidas e preocupações.

Evidências apontam que a utilização do aconselhamento em aleitamento materno no AC possibilitou o sucesso da prática de amamentação, inclusive em casos desfavoráveis ou de difícil manejo clínico^(21)^, aumentando as chances de manutenção dele em sua forma exclusiva até os quatro^(9,22)^ e seis meses^(9,23)^ e reduzindo as chances do uso de fórmulas durante a internação no AC^(24)^. Ainda, estudos apontam que o empoderamento da nutriz por meio do aconselhamento reduziu a crença da produção insuficiente ou leite fraco, principal causa de desmame^(25,26)^. Os resultados aqui alcançados avançam as evidências e sinalizam a relevância no processo de tornar-se nutriz para mulheres primíparas.

Contudo, mesmo diante das evidências, e de ser uma estratégia descrita e altamente recomendada pela OMS, a prática é pouco comum, considerando que mais da metade da população mundial não possui acesso pleno a serviços essenciais de saúde, incluindo o acesso ao aconselhamento por profissionais capacitados^(27)^, o que impacta negativamente o apoio recebido por mulheres durante a amamentação.

Ressalta-se que metade das mulheres lactantes tem dificuldade com o aleitamento materno nos três primeiros dias após o parto, necessitando de suporte^(28)^. Ainda, estudo apontou que as primíparas têm mais dificuldade em iniciar o aleitamento materno do que as multíparas, atrasando seu início^(29)^, que pode comprometer o sucesso da amamentação, e os resultados aqui obtidos sinalizam a aposta no aconselhamento em aleitamento materno no AC como um contraponto. Isto se deve, sobretudo, pelo alcance relacional obtido, no qual a pessoa se sente confortável e acolhida na presença ofertada pelo aconselhador. O comportamento do aconselhador se revela essencial para o ato de aconselhar.

Em contraponto, insistências familiares sobre uso de bicos e mamadeiras, sobretudo quando há dependência da mulher de sua rede familiar para apoio aos cuidados da criança quando vai trabalhar, colocam em risco a exclusividade da amamentação, conforme emergiu nas falas, independentemente do aconselhamento.

Os estudos apresentados apontam a importância do suporte para primíparas e a efetividade da estratégia do aconselhamento em aleitamento materno para superação de dificuldades e manutenção de sua exclusividade conforme recomendação.

A possibilidade de desmame se apresenta diante de intercorrências e dificuldades. Realça-se a fala de uma participante que teve uma intercorrência pós-parto e que o aconselhamento foi insuficiente para a manutenção da amamentação, pois era necessária uma filosofia institucional que valorizasse e previsse a necessidade de manter a mulher como nutriz para, posteriormente, pensar em relactação.

As evidências aqui apresentadas deixaram pistas do AC como espaço diferenciado para o aconselhamento por se articular com momento particular do puerpério imediato, com indícios de exploração qualitativa neste foco. Ademais, apesar de não ter sido escopo explorado, as aconselhadoras eram enfermeiras de formação, todas com capacitação em aconselhamento, o que permite inferir exsitência de perfil de competências presentes. Assim, sugere-se desenvolvimento de estudos que se voltem às competências interacionais do aconselhador, devido à ênfase que as participantes aferiram a este foco. De todo modo, o enfermeiro está sugerido como profissional com características favorecedoras do aconselhamento em aleitamento materno.

Ademais, o vivido nas interações de aconselhamento reverbera nas memórias das mulheres, sendo reavivado diante de situações vinculadas à amamentação nas conversas internalizadas na mente, direcionando a tomada de decisão. Este aspecto longitudinal do aconselhamento é achado relevante e de avanço nas evidências existentes, provido pelo presente estudo.

### Limitações do estudo

Este estudo tem limitações, em especial relacionadas ao fato de reportar experiências de pessoas que foram expostas à intervenção de aconselhamento em aleitamento materno desenvolvida por três enfermeiras. Neste sentido, estudos que explorem o alcance do aconselhamento desenvolvido por outras categorias profissionais e em outros contextos assistenciais podem compor os achados desta investigação.

As entrevistas realizadas remotamente podem ser consideradas como fragilidade do estudo; no entanto, o uso de câmeras pelas entrevistadoras e o investimento na criação e um contexto relacional acolhedor buscaram promover a narração e densidade dos dados. Reforça-se a importância de se desenvolver novos estudos com entrevistas presenciais; contudo, enfatiza-se que o fato de serem remotas facilitou a disponibilidade para participação.

Outra limitação do estudo refere-se ao fato de que três mulheres que compuseram o estudo piloto foram excluídas por não possuírem um *smartphone*, internet ou por ambas condições. Essa limitação compromete a generalização dos resultados, por não abarcar situações de vulnerabilidade social expressas na diversidade no acesso às tecnologias. Para sanar essa lacuna, reforça-se a importância de desenvolvimento de novos estudos que entrevistem as mulheres em seus domicílios ou em ambientes controlados.

Ademais, o estudo foi desenvolvido apenas com primíparas e em três centros hospitalares, e com pequeno número de participantes, que compromete a generalização e transferibilidade dos resultados, porém estudos qualitativos não possuem tal intenção.

### Contribuições para as áreas da enfermagem, saúde ou políticas públicas

Os resultados obtidos avançam e adensam as evidências sobre aconselhamento em aleitamento materno, sobretudo no que tange à presença profissional como elemento-chave para o relacionamento edificador da intervenção e o reverberar dessa relação na tomada de decisão em amamentação e aspectos que permeiam ele. Os relatos reforçam a importância da inserção do aconselhamento em aleitamento humano na formação profissional, no dimensionamento de pessoal e nas políticas públicas de promoção e proteção à amamentação. Ademais, enfatiza-se a importância do enfermeiro como agente altamente relevante para ações de aconselhamento, conforme evidenciado no estudo.

## CONSIDERAÇÕES FINAIS

As mulheres apontaram elementos dos pilares essenciais do aconselhamento em aleitamento materno e sua influência na tomada de decisão pela manutenção do aleitamento materno exclusivo no primeiro ano de vida das suas crianças, com destaque para os efeitos do comportamento da presença das enfermeiras aconselhadoras para a relação de cuidado e seu alcance.

## Data Availability

Os dados da pesquisa estão disponíveis no corpo do artigo.
